# Measurement of Waist and Hip Circumference with a Body Surface Scanner: Feasibility, Validity, Reliability, and Correlations with Markers of the Metabolic Syndrome

**DOI:** 10.1371/journal.pone.0119430

**Published:** 2015-03-06

**Authors:** Lina Jaeschke, Astrid Steinbrecher, Tobias Pischon

**Affiliations:** Molecular Epidemiology Group, Max Delbrueck Center for Molecular Medicine (MDC), Berlin, Germany; University College London, UNITED KINGDOM

## Abstract

**Objective:**

Body surface scanners (BS), which visualize a 3D image of the human body, facilitate the computation of numerous body measures, including height, waist circumference (WC) and hip circumference (HC). However, limited information is available regarding validity and reliability of these automated measurements (AM) and their correlation with parameters of the Metabolic Syndrome (MetS) compared to traditional manual measurements (MM).

**Methods:**

As part of a cross-sectional feasibility study, AM of WC, HC and height were assessed twice in 60 participants using a 3D BS (Vitus^smart^XXL). Additionally, MM were taken by trained personnel according to WHO guidelines. Participants underwent an interview, bioelectrical impedance analysis, and blood pressure measurement. Blood samples were taken to determine HbA1c, HDL-cholesterol, triglycerides, and uric acid. Validity was assessed based on the agreement between AM and MM, using Bland-Altman-plots, correlation analysis, and paired t-tests. Reliability was assessed using intraclass correlation coefficients (ICC) based on two repeated AM. Further, we calculated age-adjusted Pearson correlation for AM and MM with fat mass, systolic blood pressure, HbA1c, HDL-cholesterol, triglycerides, and uric acid.

**Results:**

Body measures were higher in AM compared to MM but both measurements were strongly correlated (WC, men, difference = 1.5cm, r = 0.97; women, d = 4.7cm, r = 0.96; HC, men, d = 2.3cm, r = 0.97; women, d = 3.0cm; r = 0.98). Reliability was high for all AM (nearly all ICC>0.98). Correlations of WC, HC, and the waist-to-hip ratio (WHR) with parameters of MetS were similar between AM and MM; for example the correlation of WC assessed by AM with HDL-cholesterol was r = 0.35 in men, and r = -0.48 in women, respectively whereas correlation of WC measured manually with HDL cholesterol was r = -0.41 in men, and r = -0.49 in women, respectively.

**Conclusions:**

Although AM of WC, HC, and WHR are higher when compared to MM based on WHO guidelines, our data indicate good validity, excellent reliability, and similar correlations to parameters of the MetS.

## Introduction

Obesity is an established risk factor for increased morbidity and mortality [1–6]. The assessment of obesity is primarily based on the body mass index (BMI, the weight in kilograms divided by the square of the height in meters); however, large studies conducted over the past years have shown that body fat distribution contributes to morbidity and mortality beyond the degree of obesity per se. Thus, abdominal obesity is more closely associated with risk of morbidity and mortality than is gluteofemoral obesity. Hence waist circumference (WC) or the waist-to-hip ratio (WHR), as indicators of abdominal obesity, may be better predictors of risk than the BMI for several diseases, including cardiovascular disease (CVD), cancer, type 2 diabetes, and the Metabolic Syndrome (MetS). WC and hip circumference (HC) are usually assessed in manual measurements (MM) with flexible but non-stretchable tapes according to guidelines by the World Health Organization (WHO) at the midpoint between the last rib and the iliac crest, and at the level of the largest lateral extension of the hips, respectively, both in a horizontal plane [7]. However, standardized precise and accurate measurement of WC and HC is often challenging, time consuming, and may require assistance, particularly in obese individuals [8–12].

With the technical development of three-dimensional (3D) body surface scanners (BS) to assess body shape for the clothing industry, an attractive alternative automated measurement (AM) may have become available [13], particularly for large-scale epidemiological studies, where standardized, fast, accurate, and precise assessment methods are key for valid estimation of disease risk. Recently, measurement of body size has been conducted for the clothing industry in more than 13 000 individuals using a laser-based BS (Vitus^smart^XXL, Human Solutions GmbH, Kaiserslautern, Germany) [13]. Within 12 seconds, that BS scans the body surface, produces a 3D image, and is capable to calculate 153 body size measures for the clothing industry. However, it is currently unclear to what extent WC and HC measurements derived from this AM agree with traditional MM based on WHO guidelines, and what their correlations are with parameters of the MetS. Such information, however, is important because high correlations with parameters of the MetS are indicative that such measurements appropriately characterize the “obesity phenotype” that is relevant for disease risk. In addition, it is unclear to what extent measurements with a 3D BS are feasible in epidemiological studies.

The aim of the present study was therefore to examine the agreement of WC, HC and height measurements derived from AM using the Vitus^smart^XXL BS as compared to MM, as well as the reliability of AM. In addition, we examined correlations with markers of MetS [14] and the feasibility of using AM in an epidemiological setting.

## Methods

### Study population

Data in this cross-sectional study were collected from September until December in 2012 at the study center Berlin-North during a feasibility study for the German National Cohort (GNC, a population based cohort study), which aimed to include at least 100 participants. The original objective of the feasibility study was to build up personnel and technical infrastructure for the GNC, to establish recruitment and study procedures as well as to investigate the applicability of protocols and methods. Representativeness was no aim of these feasibility studies. Participants were recruited based on randomly selected addresses received from municipal registries in the Northern part of Berlin and adjacent communities in the State of Brandenburg, stratified by gender (50:50) and age based on a standardized recruitment protocol. Inclusion criteria were age 20–69 years, German language skills, and the ability to give informed consent. To ensure the scheduled number of participants within short time, 799 people were contacted of which 239 agreed to participate. 109 persons were finally included in the feasibility study of the German National Cohort. Since the body scanner has been only available since October 2012, only 63 of these could be asked to also participate in an AM using the 3D BS.

The study protocol was approved by the ethics committee of the Charité—Universitätsmedizin Berlin and the local data protection officer. All participants gave written informed consent.

### Data collection

Information on socio-demography, economic and lifestyle characteristics and pre-existing medical conditions were collected as part of a standardized personal interview. Participants were asked to report their age, smoking status, frequency of alcohol intake, education, occupation, and if they had ever been diagnosed with diabetes mellitus or with elevated blood lipid levels by a physician.

MM of anthropometry was taken by trained personnel with participants wearing only light underwear. Body height (in cm) and weight (in kg) were measured with the measuring station SECA 285 (SECA, Hamburg, Germany), WC and HC (both in cm) with the tape measure SECA 201 (SECA, Hamburg, Germany) according to WHO guidelines [15]. Height was missing in one participant.

**Fig 1:**
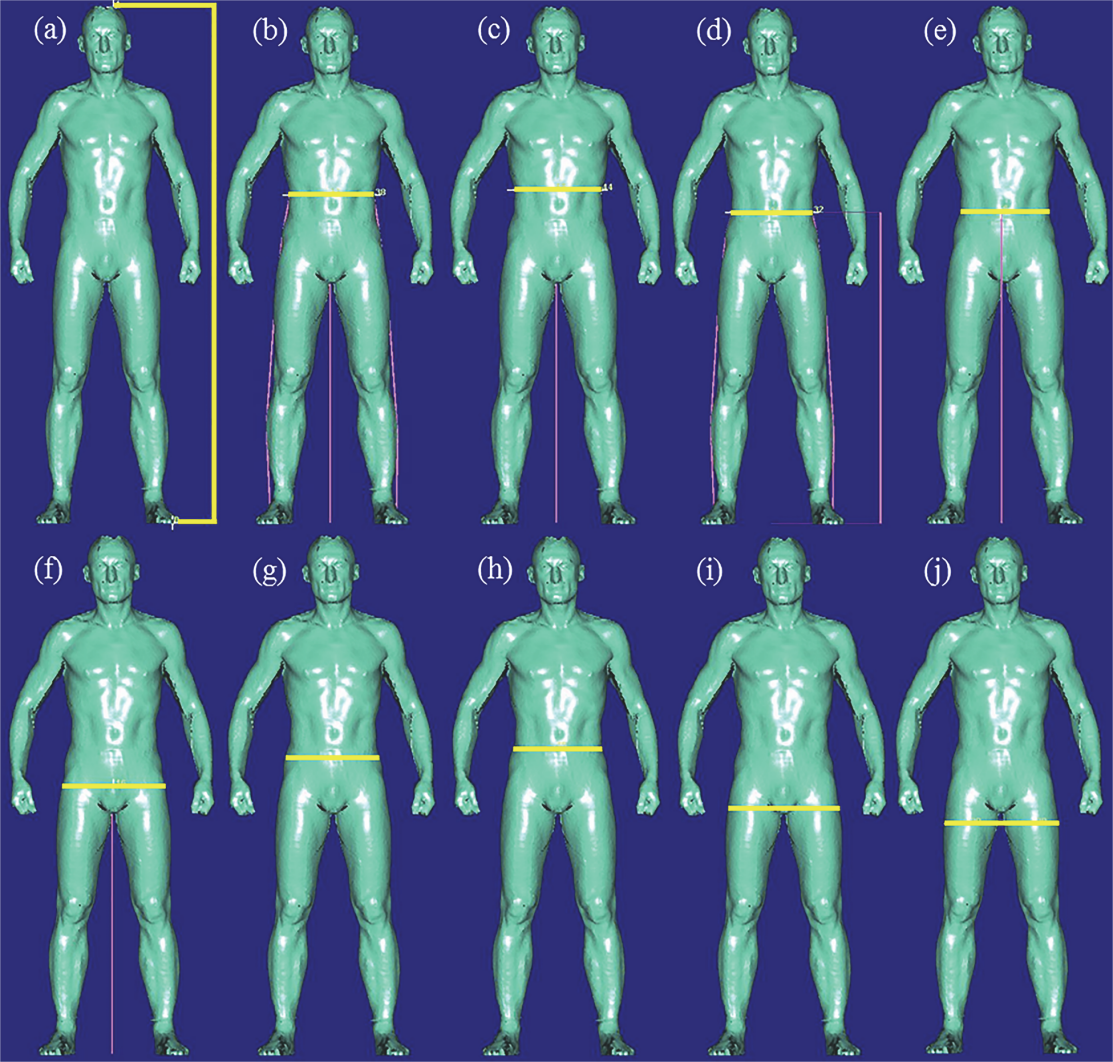
3D scan images (screenshots from the AnthroScan Professional software interface) with marked measures as assessed in this study. Yellow lines indicate the actual measuring site; purple lines indicate reference measurements. (a) body-height; (b) waist-girth, (c) high-waist-girth, (d) waist-band, (e) belly-circumference; (f) buttock-girth, (g) middle-hip, (h) high-hip-girth, (i) hip-girth, and (j) hip-thigh-girth (Picture: modified from Human Solutions GmbH)

AM was performed using the BS Vitus^smart^XXL and the software AnthroScan Professional (both Human Solutions GmbH, Kaiserslautern, Germany) with participants being undressed up to the underwear, wearing a bathing cap, and standing in a standard position defined by the manufacturer (standing upright with legs hip-wide apart, arms slightly bend and away from the body, hands making a fist with thumbs showing forward, and head positioned in accordance with the Frankfort Horizontal). Using four eye-safe lasers and eight cameras, the BS provides a 3D point cloud based on optical triangulation. From this, 153 anthropometric measures are computed by the BS software according to ISO 20685:2005 [16]. These include four parameters for WC (*waist-girth*, *high-waist-girth*, *waist-band*, *belly-circumference)*, five parameters for HC (*buttock-girth*, *middle-hip*, *high-hip-girth*, *hip-girth*, *hip-thigh-girth*), and one parameter for WHR (based on *waist-girth* and *buttock-girth)*, which were used in the following analyses (**Fig. 1**). All participants were scanned twice, while breathing normally in scan 1 and breathing out in scan 2. Based on a predefined checklist (**S1 Table**), 3D pictures were controlled visually with regard to their quality, deviations from the standard protocol, and plausibility of measuring points and measured values. We assessed number of scans completed and pictures acquired. For feasibility purpose we further assessed duration of AM, deviations from the standard posture with regard to arm and leg posture and improper clothing, as well as the participants’ burden due to AM (no, little, moderate or high burden) and number of required scan attempts for yielding an evaluable picture.

After an initial rest of 5 minutes three sitting blood pressure measurement were taken with intervals of 2 minutes. Measurement was performed using the blood pressure gauge HEM 705IT (OMRON, Mannheim, Germany) and a cuff size suitable to the upper arm width. Since it is known that the first measurement is affected by adaptation to the sitting condition, we used the mean of the second and third systolic blood pressure (SBP) measurement for analysis [17].

Blood samples were collected and HbA1c, HDL-cholesterol (HDL-C), triglycerides, and uric acid were determined as parameters of the MetS [14]. Time since last meal at blood draw is provided in Table 1. Laboratory analysis was performed by the hospital Laborverbund Brandenburg-Berlin GmbH (Berlin, Germany).

**Table 1:** Characteristics of the study population (n = 59).

	men		women	
	n	%	n	%
N	27	100.0	32	100.0
age classes				
20–39 years	4	14.8	5	15.6
40–49 years	5	18.5	7	21.9
50–59 years	7	25.9	12	37.5
60–69 years	11	40.7	8	25.0
smoking status				
never-smokers	13	48.2	13	40.6
smokers	9	33.3	8	25.0
former smokers	5	18.5	11	34.4
alcohol consumption				
never	1	3.7	2	6.3
max. 1x/ month	4	14.8	11	34.4
2–4x/ month	8	29.6	14	43.8
2–3x/ week	9	33.3	3	9.4
4x/ week or more frequently	5	18.5	2	6.3
school education				
without qualification for university entrance	17	63.0	20	62.5
with qualification for university entrance	10	37.0	12	37.5
occupation				
full time	18	66.7	15	46.9
part time	0	0.0	7	21.9
not employed	9	33.3	9	28.1
diabetes mellitus	2	7.4	1	3.1
elevated blood lipid levels	11	40.7	7	21.9
time since last meal at blood draw				
< 3 hours	20	74.1	24	75.0
3–6 hours	6	22.2	7	21.9
> 6 hours	1	3.7	1	3.1
	**mean**	**SD**	**mean**	**SD**
BMI, kg/m^2^	26.6	3.2	25.7	4.6
relative fat mass, %kg	24.4	7.2	36.2	6.5
systolic blood pressure, mmHg	133.2	15.1	127.5	18.2
diastolic blood pressure, mmHg	80.9	9.4	80.2	9.9
HbA1c, mmol/mol	38.5	5.3	37.7	5.9
HDL cholesterol, mg/dl	52.7	16.1	63.2	14.3
Triglycerides, mmol/l *	2.0	1.6–2.5	1.4	1.1–1.7
uric acid, mg/dl	5.9	1.2	4.1	1.0

BMI, body mass index; HbA1c, hemoglobin A1c; HDL-C, high density lipoprotein cholesterol; SD, standard deviation

*geometric mean and 95%-CI.

Body composition was assessed using bioelectrical impedance analysis (BIA) carried out with participants undressed up to the underwear using the measuring station SECA 515 (SECA, Hamburg, Germany). Relative fat mass (percent of body weight; %FM) was determined.

### Statistical analysis

Normally distributed data are presented as mean ± standard deviation (SD); not-normally distributed data (triglycerides) were log-transformed and are presented as geometric mean and 95%- confidence interval (CI). Data on age (20–39, 40–49, 50–59, 60–69 years), smoking status (never, current or past), alcohol intake frequency (never, ≤1x/month, 2–4x/month, 2-3/week, ≥4x/week), education (with/without qualification for university entrance), and occupation (full time, part time or not employed) were categorized. Furthermore we used WHO cut-offs [15] to categorize WC (men, WC≤/>0.94; women, WC≤/>0.80) and WHR (men, WHR</≥0.90; women, WHR</≥0.85).

Agreement of height, WC, HC and WHR between MM and AM was assessed using Bland-Altman analysis [18,19] and by calculating Pearson correlation coefficients. To ensure variance homogeneity, correlation analysis was also conducted between the difference of both methods and their mean. Mean differences between MM and AM were examined using t-tests for two dependent samples. Further, the degree of re-classification of categorized WC and WHR (“below/above limit”) according to WHO guidelines [15] between MM and AM was examined by calculating kappa coefficients (κ) [20]. κ was assessed according to Altman’s reference range: 0.41–0.60 = moderate agreement; 0.61–0.80 = good agreement [20]. In addition, percentage of re-classified WC and WHR in relation to all participants was calculated.

Reliability of AM was assessed using intraclass correlation coefficients (ICC) based on the repeated AM. The validity of AM was examined indirectly by means of age-adjusted partial correlation analysis (Pearson) for WC and WHR of MM and AM (4 mentioned waist measures) with %FM, SBP and blood concentration of HbA1c, HDL-C, triglycerides, and uric acid.

All data are presented stratified by gender. P-values presented are 2-tailed and p<0.05 were considered statistically significant. Analyses were performed using SAS Enterprise Guide, version 4.3 (SAS Institute Inc, Cary, NC).

## Results

Out of the 63 persons who were asked to participate in the body surface scanner examination, two persons refused and one person had to be excluded because of technical problems with the scanner. Thus, 60 participants (27 men and 33 women) underwent AM. We acquired two scans from all 60 participants yielding a total of 120 3D images, which were checked visually. Overall, their quality, as assessed by completeness of the point cloud, was good. Deviations from the standard posture were found in 15.0% (5.8% for arm postures and 9.2% for leg postures). In all these cases arm and leg posture was closer to the body than defined in the standard protocol. Nevertheless, these deviations did not affect calculation of height, WC, HC or WHR. One woman wore an undershirt and no bathing cap, resulting in incomplete 3D pictures that did not allow automated calculation of body height, HC, and WC. One man wore an undershirt, which did not allow calculation of WC and WHR. Additionally, 13 participants (21.7%) also wore undershirts or underpants but since they were tightly fitted, they did not affect AM. We thus had 59 participants with information about HC, and 58 participants with information on WC and WHR; both from two replicate scans.

Participants reported no (98.2%) or little (1.8%) burden of AM. Median time required for AM was 10.0 minutes (interquartile range, IQR 7.0–23.0 minutes) which is comparable to the time required for MM. Most scans (97.6%) were successfully performed at the first attempt; in 2.4% cases a second scan had to be performed. The participants’ socio-demographic lifestyle and metabolic characteristics are summarized in **Table 1**.

**Fig 2:**
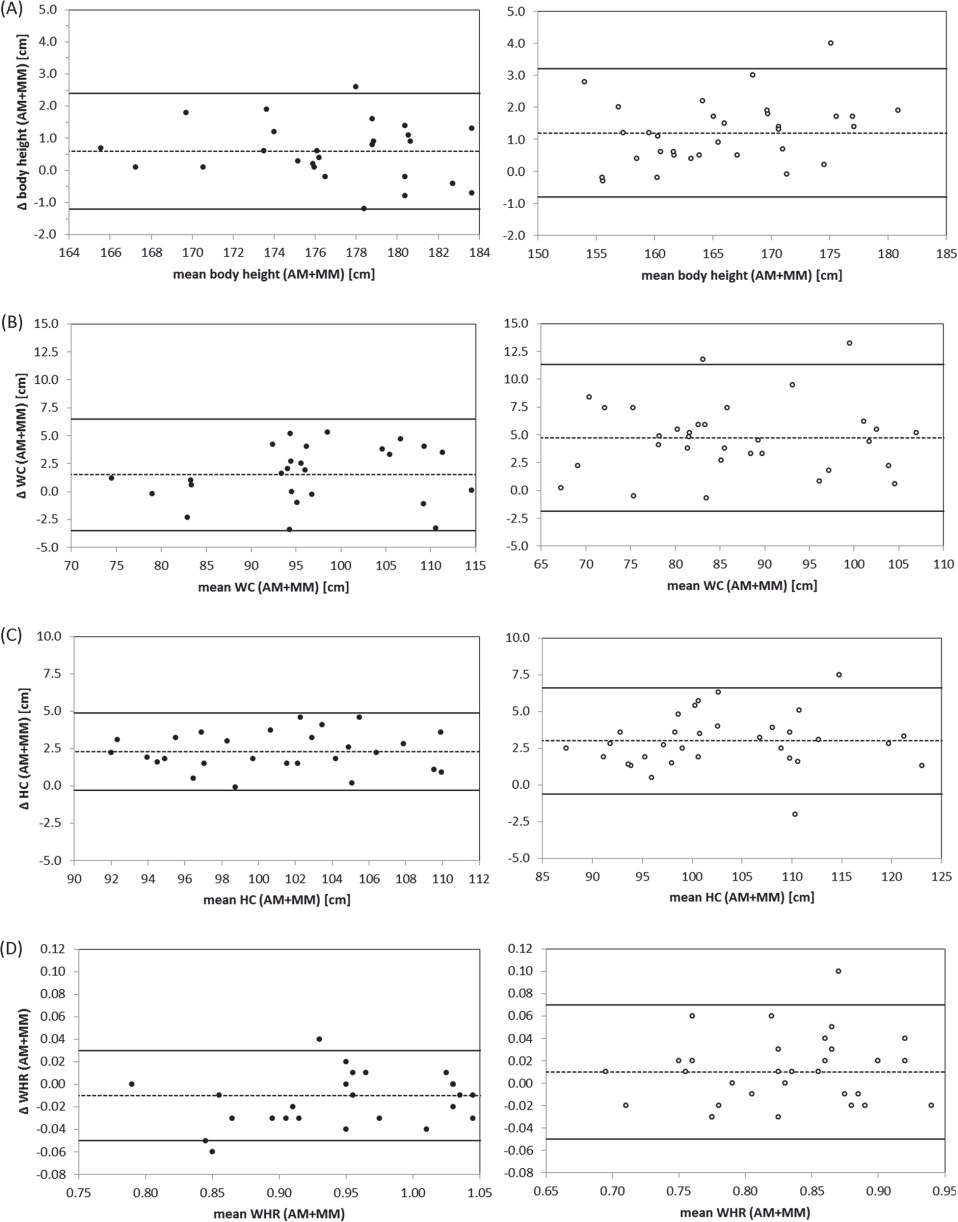
Bland-Altman-plots for comparison between MM and AM body height (panel A), WC (panel B), HC (panel C), and WHR (panel D) for men (filled circle) and women (light circle). For WC, MM was compared to the corresponding AM *waist-girth*, for HC to the corresponding AM *buttock-girth*. AM WHR was calculated using the measures *waist-girth* and *buttock-girth*. Differences (Δ) between methods were calculated as AM-MM.

Comparing anthropometric measures acquired by AM and MM, we found strong correlations for height between the two methods (men, r = 0.98; women r = 0.99, **Table 2**). However, AM provided significantly larger body heights compared to MM (**Fig. 2**). The mean differences between the two methods were d = 0.6±0.9cm (p = 0.003) and d = 1.2±1.0cm (p<0.0001) for men and women, respectively. The within-person differences between the AM and the MM were not significantly correlated with the within-person means of AM and MM (men, r = -0.15; women, r = 0.31).

**Table 2:** Comparison of manual (MM) and automated anthropometry (AM).

	manual anthropometry (MM)		automatedanthropometry (AM)		difference AM-MM		t-test AM vs. MM	correlation AM with MM	correlation difference (AM-MM) with mean (AM+MM)
		mean		mean	mean	SD	p	r	r
**men** (n = 27)	**body height, cm**	176.5	**body-height, cm**	177.0	0.6	0.9	**0.003**	**0.98**	-**0.15**
	**waist, cm**	96.1	**waist-girth, cm**	97.3	1.5	2.5	**0.005**	**0.97**	**0.17**
			**high-waist-girth, cm**	96.4	0.7	2.8	0.24	0.96	0.003
			waist-band, cm	95.8	0.0	4.6	0.98	0.91	-0.57
			belly-circumference, cm	99.6	3.8	2.8	<0.0001	0.96	-0.25
	**hip, cm**	99.8	**buttock-girth, cm**	102.1	2.3	1.3	**<0.0001**	**0.97**	**0.06**
			middle-hip, cm	99.8	-0.1	4.7	0.94	0.86	0.67
			high-hip-girth, cm	100.0	0.1	5.7	0.90	0.83	0.71
			hip-girth, cm	102.9	3.1	1.6	<0.0001	0.96	-0.02
			hip-thigh-girth, cm	94.8	-5.0	4.2	<0.0001	0.65	-0.25
	**WHR**	0.96	WHR*	0.95	-0.01	0.02	**0.006**	**0.95**	**0.26**
									
**women** (n = 32)	**body height, cm**	165.3	**body-height, cm**	166.5	1.2	1.0	**<0.0001**	**0.99**	**0.31**
	**waist, cm**	84.3	**waist-girth, cm**	89.0	4.7	3.3	**<0.0001**	**0.96**	**0.03**
			**high-waist-girth, cm**	85.6	1.3	3.8	0.07	0.94	-0.02
			waist-band, cm	91.8	7.5	4.1	<0.0001	0.94	-0.54
			belly-circumference, cm	93.6	9.2	3.4	<0.0001	0.95	-0.29
	**hip, cm**	101.9	**buttock-girth, cm**	104.8	3.0	1.8	**<0.0001**	**0.98**	**0.10**
			middle-hip, cm	100.1	-1.8	5.1	0.06	0.87	0.26
			high-hip-girth, cm	95.5	-6.4	4.9	<0.0001	0.87	0.17
			hip-girth, cm	106.4	4.5	2.5	<0.0001	0.97	0.20
			hip-thigh-girth, cm	97.6	-4.2	5.6	<0.0001	0.80	-0.14
	**WHR**	0.83	WHR*	0.84	0.01	0.03	**0.04**	**0.89**	**0.14**

AM, automated measurement; MM, manual measurement; SD, standard deviation; WHR, waist-to-hip-ratio;

*calculated from waist-girth and buttock-girth.

All of the four acquired automated waist measurements (*waist-girth*, *high-waist-girth*, *waist-band*, *belly-circumference)* were highly correlated with manual waist measurement (range of r, men 0.91 to 0.97; women 0.94 to 0.96). Nevertheless, measurements of WC were generally higher in AM compared to MM (range in mean difference, men 0.0 to 3.8; women 1.3 to 9.2). *Waist-girth* had the lowest SD of the four AM waist measurements and highest correlation with MM (men, r = 0.97; women, r = 0.96). *High-waist-girth* showed a comparable correlation to the manual WC measurement (men, r = 0.96; women, r = 0.94) and a not significant mean difference from MM (men, d = 0.7±2.8cm, p = 0.24; women, d = 1.3±3.8cm, p = 0.07). We found evidence of heteroscedasticity for *waist-band* (men, r = -0.57; women, r = -0.54). Measures of HC were also highly correlated between the two methods (range of r, men 0.65 to 0.97; women 0.80 to 0.98). However, we found systematic mean differences between MM and AM (range in mean difference, men -5.0 to 3.1; women -6.4 to 4.5). *Buttock-girth* showed the highest correlation with MM (men, r = 0.97; women, r = 0.98) and the lowest SD from MM, but was significantly larger than MM (men, d = 2.3±1.3cm, p<0.0001; women, d = 3.0±1.8cm, p<0.0001). Similar values were determined for *hip-girth* (men, r = 0.96, d = 3.1±1.6cm, p<0.0001; women, r = 0.97, d = 4.5±2.5cm, p<0.0001). Men showed heteroscedasticity for *middle-hip* (r = 0.67) and *high-hip-girth* (r = 0.71). WHR was significantly smaller for AM than MM for men (d = -0.01±0.02, p = 0.006) but larger for women (d = 0.01±0.03, p = 0.04). Variance was distributed homogeneously (men, r = 0.26; women, r = 0.14).

Classifying participants into high/low risk category according to WHO guidelines for WC or WHR and comparing the classification based on waist girth as assessed by AM with that of MM we found moderate agreement for WC (men, κ = 0.47; women κ = 0.46) and good agreement for WHR (men, κ = 0.79; women κ = 0.75) (**Table 3**). For WC, 23.1% (n = 6) of men and 25% (n = 8) of women, respectively, were in discordant categories when classified based on AM or MM. Five of six men and all women, who were re-classified, changed into the “above limit” category when analyzing AM. For WHR, 7.7% (n = 2) of men and 12.5% (n = 4) of women were in discordant categories. All men changed from the “above limits” to the “below limits” category when analyzing AM, for women the opposite was true.

**Table 3:** Agreement in the classification of the waist circumference and WHR between the manual and the automated measurement.

		below/above limit	agreement classification AM vs. MM	re-classification
	limit	N	kappa	%
**men** (n = 26)				
**WC MM, cm**	≤/> 94 cm	10/16	0.47	23.1
**WC AM, cm**	≤/> 94 cm	6/20		
**WHR MM, cm**	</≥ 0.90	5/21	0.79	7.7
WHR AM*, cm	</≥ 0.90	7/19		
**women** (n = 32)				
**WC MM, cm**	≤/> 80 cm	14/18	0.46	25.0
**WC AM, cm**	≤/> 80 cm	6/26		
**WHR MM, cm**	</≥ 0.85	20/12	0.75	12.5
WHR AM*, cm	</≥ 0.85	16/16		

AM, automated measurement; MM, manual measurement; WC, waist circumference; WHR, waist-to-hip-ratio;

*calculated from waist-girth and buttock-girth.

Correlations of WC and WHR as assessed by AM and MM with metabolic characteristics are shown in **Table 4**. Strength and direction of the correlations with %FM, SBP, HbA1c, HDL-C, triglycerides, and uric acid were similar for *waist-girth* (men, r = 0.85, 0.04, 0.17, -0.35, -0.06, 0.22; women, r = 0.87, 0.20, 0.32, -0.48, 0.44, 0.59, respectively) when compared to the corresponding WC of MM (men, r = 0.84, 0.03, 0.19, -0.41,0.06, 0.20; women, r = 0.82, 0.20, 0.32, -0.49, 0.37, 0.62, respectively). The analogue correlations of *high-waist-girth*, *waist-band*, and *belly-circumference* were comparable to those of *waist-girth* and MM. Correlations of WHR with %FM, SBP, HbA1c, HDL-C, triglycerides, and uric acid were also similar for AM (men, r = 0.71, 0.26, 0.28, -0.21, -0.09, 0.16, respectively; women, r = 0.62, 0.11, 0.34, -0.68, 0.66, 0.38, respectively) when compared to MM (men, r = 0.77, 0.25, 0.28, -0.35, 0.07, 0.12; women, r = 0.52, 0.14, 0.36, -0.62, 0.46, 0.48, respectively).

**Table 4:** Partial correlation of waist measurements and WHR according to manual and automated measurement with percent fat mass and markers of the MetS.

		partial correlation (age-adjusted)											
measuring method	measure	% of fat mass		blood pressure (systole)		HbA1c		HDL cholesterol		triglycerides (ln)		uric acid	
		r	p	r	p	r	p	r	p	r	p	r	p
**men** (n = 26)													
**MM**	waist	0.84	<0.0001	0.03	0.89	0.19	0.36	-0.41	0.04	0.06	0.80	0.20	0.34
**AM**	waist-girth	0.85	<0.0001	0.04	0.84	0.17	0.43	-0.35	0.09	-0.06	0.78	0.22	0.30
	high-waist-girth	0.87	<0.0001	0.03	0.90	0.18	0.40	-0.31	0.13	-0.09	0.66	0.21	0.32
	waist-band	0.73	<0.0001	-0.11	0.61	<0.01	1.00	-0.28	0.18	-0.09	0.67	0.08	0.71
	belly-circumference	0.81	<0.0001	0.02	0.93	0.14	0.49	-0.34	0.10	-0.06	0.79	0.19	0.37
**MM**	WHR	0.77	<0.0001	0.25	0.22	0.28	0.18	-0.35	0.09	0.07	0.74	0.12	0.27
**AM**	WHR*	0.71	0.001	0.26	0.21	0.28	0.18	-0.21	0.31	-0.09	0.69	0.16	0.45
**women** (n = 32)													
**MM**	waist	0.82	<0.0001	0.20	0.27	0.32	0.08	-0.49	0.005	0.37	0.04	0.62	<0.0001
**AM**	waist-girth	0.87	<0.0001	0.20	0.27	0.32	0.08	-0.48	0.006	0.44	0.01	0.59	<0.0001
	high-waist-girth	0.84	<0.0001	0.22	0.23	0.33	0.07	-0.46	0.009	0.40	0.03	0.62	<0.0001
	waist-band	0.86	<0.0001	0.17	0.37	0.28	0.13	-0.46	0.009	0.42	0.02	0.57	0.001
	belly-circumference	0.88	<0.0001	0.23	0.22	0.35	0.06	-0.50	0.004	0.42	0.02	0.60	<0.0001
**MM**	WHR	0.52	0.04	0.14	0.45	0.36	0.05	-0.62	<0.0001	0.46	0.009	0.48	0.006
**AM**	WHR*	0.62	0.01	0.11	0.55	0.34	0.06	-0.68	<0.0001	0.66	<0.0001	0.38	0.04

%FM, % of fat mass; AM, automated measurement; HbA1c, hemoglobin A1c; HDL-C, high density lipoprotein cholesterol; MM, manual measurement; SBP, systolic blood pressure; SD, standard deviation; WC, waist circumference; WHR, waist-to-hip-ratio;

*calculated from waist-girth and buttock-girth.

Reliability of AM was high, all ICC were >0.96, with exception of *hip-thigh-girth* in men (0.82, 95%-CI 0.65–0.91; **Table 5**). Mean differences between the two measurements were mostly positive, reflecting larger AM for scan 1.

**Table 5:** Reliability of automated measurement when using the 3D BS Vitus^smart^XXL.

	measure	measured valuescan 1	measured valuescan 2	difference scan 1–2		ICC	95%-CIfor ICC
		mean	mean	mean	SD	ICC	
**men** (n = 27)	**body-height, cm**	177.0	177.2	-0.1	0.5	1.00	0.99–1.00
	**waist-girth, cm**	97.3	96.8	0.5	1.1	0.99	0.99–1.00
	high-waist-girth, cm	96.4	95.8	0.7	1.2	0.99	0.98–1.00
	waist-band, cm	5.8	95.6	0.2	0.7	1.00	0.99–1.00
	belly-circumference, cm	99.6	99.1	0.5	1.0	0.99	0.99–1.00
	**buttock-girth, cm**	102.1	101.9	0.2	0.4	1.00	0.99–1.00
	middle-hip, cm	99.8	99.4	0.4	0.8	1.00	0.99–1.00
	high-hip-girth, cm	100.0	99.6	0.4	0.9	1.00	0.99–1.00
	hip-girth, cm	102.9	102.9	0.1	1.6	0.96	0.92–0.98
	hip-thigh-girth, cm	94.8	92.5	2.4	1.8	0.82	0.65–0.91
	WHR*	0.95	0.94	0.00	0.02	0.97	0.94–0.99
**women** (n = 32)	**body-height, cm**	166.5	166.5	0.0	0.8	0.99	0.99–1.00
	**waist-girth, cm**	89.0	89.0	0.0	2.0	0.99	0.97–0.99
	high-waist-girth, cm	85.6	85.3	0.3	2.0	0.99	0.97–0.99
	waist-band, cm	91.8	91.6	0.2	1.4	0.99	0.98–0.99
	belly-circumference, cm	93.6	93.2	0.4	1.4	0.99	0.98–1.00
	**buttock-girth, cm**	104.8	104.7	0.1	0.7	1.00	0.99–1.00
	middle-hip, cm	100.1	100.1	-0.1	1.4	0.99	0.98–1.00
	high-hip-girth, cm	95.5	95.1	0.4	1.4	0.99	0.98–1.00
	hip-girth, cm	106.4	106.1	0.3	1.1	0.99	0.99–1.00
	hip-thigh-girth, cm	97.6	96.7	0.9	1.2	0.98	0.97–0.99
****	WHR*	0.84	0.84	0.00	0.02	0.96	0.92–0.98

ICC, intraclass correlation coefficient; SD, standard deviation; WHR, waist-to-hip-ratio;

*calculated from waist-girth and buttock-girth.

## Discussion

Our study showed good technical and practical feasibility of AM. AM was highly and significantly correlated with MM, but provided larger circumferences and body heights compared to MM. We found excellent reliability, and evidence for a good validity of AM. Even in case of deviations from the standard protocol, AM data were interpolated mostly completely. This ensures a safe data collection in epidemiological surveys, where not all participants will be capable of holding the standard posture due to age or physical constitution.

We found strong correlations between AM and MM for body height, but AM resulted on average in significantly larger values compared to MM. This was found for another BS type, too [21]. This finding is unexpected, since participants are scanned with the legs hip-wide apart to enable the identification of the crotch as a reference point of the BS software, which is in contrast to the MM based on WHO guidelines, with legs closed. The most possible explanation may be that during AM participants often may not meet the Frankfurt horizontal plan as defined in the guidelines for MM. In MM, participants have fixed orientation points (measuring station, vernier caliper) and can be guided into the correct posture by the investigator. During AM they are freestanding, which may result in partly incorrect head and body posture. In a study investigating postural deviations between two repeated AM 24h apart, it was shown that complying to the Frankfurt Horizontal is weakly reliable during AM, with large random, intraindividual and postural error [22]. A visual fixation scale, adapted to the participant’s height, might support a correct head posture. The current measuring procedure should be optimized, since overestimating body height may result in underestimating BMI and thus in invalid risk classification.

The strength of correlation of the automated measures *waist-girth* and *high-waist-girth* with the manually measured waist was similar to those observed in other studies [23,24]. This may not seem too surprising since *waist-girth* is measured at the midpoint between the lowest rib and the iliac crest, which is in accordance with the WHO guideline for MM of WC [15]. However, this finding is still remarkable because it implies that the software is apparently able to appropriately identify skeletal reference points, even for obese people. In comparison to *waist-girth* and *high-waist-girth*, w*aist-band* and *belly-circumference* showed either a weaker correlation with MM, a higher SD, heteroscedasticity, or a combination thereof. Furthermore, *waist-band* is an apparel measure which drops ventrally and thus does not comply with the definition of the MM. In accordance with other studies using the Vitus^smart^XXL [25–27] or other scan types [21,28] we found significantly larger WC for all AM measures, when compared to MM. These findings appeared to be explained by the risk of tissue constriction and incorrect alignment of the tape measure during MM. The latter could be shown in several studies, with high intra- and interindividual variances for repeated waist MM [10,12,27]. Further, participants might tend to hold their breath and pull in their stomach due to the contact during MM, either reflexively or consciously. Additionally, studies demonstrate that the arm posture can significantly influence the WC [9,29]. During AM in our study, the arm position was fixed so that it neither shadows the torso nor stretches the waist area. If this was not so during MM, stretching the abdomen could have biased WC measuring. All these aspects could result in an underestimation of WC during MM and may explain the observed mean difference. We speculate that using AM may avoid possible measurement errors that may typically occur during MM of WC.

Of the five automated hip measurements, we found strong correlations with MM for *buttock-girth* and *hip-girth*. Wells et al. reported a similar strength of correlation [24]. The other three 3D hip measures were weaker correlated with MM, had a higher SD, and/or tended to heteroscedasticity. Further, the mean difference between AM and MM of these three were negative, indicating that AM is smaller than MM, which is not plausible, as described below. Significantly larger HC for AM than for MM were observed in other studies, too [21,25,27,28]. Again, tissue constriction, tensing the gluteal muscle, or not measuring at the correct point could underestimate the HC. Most relevant might be the default leg posture during AM, which is hip-width apart, whereas it was demonstrated that a wider leg position results in significantly larger HC [30]. Manual HC is measured with legs closed, which could plausibly explain larger HC for AM than for MM. Since the overestimation of the HC results in underestimating the WHR, the software’s algorithm should be developed further to correct the HC and enable a valid risk prediction.

Compared to MM we found in AM significantly smaller WHR in men and significantly larger WHR in women. These findings may be explained by the observed larger mean differences of WC (i.e. *waist-girth*) between MM and AM for women than for men. Similar observation were made by Heuberger et al., who compared AM and MM in a female collective and reported a 16.0% change of WHR classification when using AM instead of MM, which is similar to our findings [27].

We observed correlations between the markers of the MetS and % fat mass with WC and WHR, and we found only small differences between AM and MM in strength and significance of correlation of MetS parameters and % fat mass with WC and WHR. These findings are in accordance with one other study [31]. Thus, data from AM are to a similar extent as from MM valid parameters in the metabolic characterization. The applicability of the BS Vitus^smart^XXL for risk profiling was demonstrated by Petrescu et al., who recently showed, that a large hip-to-waist-ratio, determined using AM, is a protective factor for DM [32]. Although the association of SBP and anthropometric circumferences is intensively described [33–36], we found only very weak correlations for both measuring method. This might be explained by the fact, that we had no information on antihypertensive medication.

We found high reliability for all AM, which is consistent with published data [23,28]. These data indicate that a single measurement of WC, HC, or WHR using AM is sufficient in large scale epidemiological studies. Further the high reliability of AM indicates a reasonable robustness of the method even if small deviations from the standard posture might occur. These data suggest that the differences between AM and MM observed in our study likely do not result from poor reliability of AM. Interestingly, even for WC reliability of AM was high despite the fact that participants breathed normally in scan 1 and exhaled in scan 2, indicating that reliability is obviously not substantially affected by breathing. Participants in our study might generally have breathed flatly in order not to blur the pictures, explaining the little differences in WC between normal breathing and exhaling.

The high reliability of AM, combined with the high correlations of AM with MM suggests that the relative ranking of individuals based on WC, HC, WHR, or height, e.g. based on percentiles, is likely not different between AM or MM, and will not influence the strengths of associations with disease outcomes (e.g., relative risk estimates). However, the systematic differences observed in our study suggest that categorization of individuals based on absolute cut-offs for WC, HC or WHR may result in misclassification when using AM in comparison to MM. In order to avoid such misclassification, cut-offs need to be revised for AM to enable valid risk estimation in epidemiological studies.

The sample size of our study was relatively small and did not aim to be representative of the general population; therefore, our results regarding the acceptance of AM need to be interpreted cautiously. However, the participants’ characteristics in our study were quite broad, and the results for validity and reliability of AM found in our study should be similar to other more general populations with similar characteristics. Nevertheless, further studies are warranted to examine the validity and reliability in subjects with different phenotypes, e.g. persons with extreme obesity or diseased populations. The measurement conditions between the two AM per participant that were used to assess reliability differed slightly; however, the intraclass correlation coefficients were close to 1, indicating excellent reliability despite this methodological limitation. Nevertheless, we cannot rule out the possibility that measures assessed with the body scanner that were not the subject of our analyses are affected by breathing. Most participants in our study did not provide fasting blood samples, which may affect TG concentrations. Nevertheless, we expect any misclassification in TG levels to be non-differential, and, therefore, the correlation coefficients found for TG are likely underestimates of the true correlation coefficients.

Strength of our study is the cross-sectional design with the simultaneous assessment of anthropometric measures with both methods. Thus, observed differences are attributable to differences between the methods and not due to changes in the person’s anthropometry over time. Further, MM was done by highly trained and experienced investigators, and all investigations were performed based on standardized protocols.

In conclusion, our study shows that AM of WC, HC, and WHR using a AM BS are higher when compared to MM based on WHO guidelines; however, our data indicate good validity, excellent reliability, and similar correlations to parameters of the MetS of AM when compared to MM. AM using 3D BS may thus be a good alternative method for fast, reliable and standardized assessment of WC, HC, and WHR in large scale epidemiologic studies.

## Supporting Information

S1 Table Criteria for the visual checking of the 3D images. (DOCX)
