# Supplementary material for: Measurement of Waist and Hip Circumference with a Body Surface Scanner: Feasibility, Validity, Reliability, and Correlations with Markers of the Metabolic Syndrome
Source: PLoS One. 2015 Mar 6;10(3):e0119430. doi: 10.1371/journal.pone.0119430 (PMC4352076; doi:10.1371/journal.pone.0119430)
Supplement: S1 Table — (DOCX) [file pone.0119430.s001.docx]

Table S1: Criteria for the visual checking of the 3D images

|  |
| --- |
| Did the participant wear clothes other than light underwear? |
| *if yes, which?* |
| Was the bathing cap used? |
| Was the body posture according to SOP? |
| *if not, what is deviate from SOP?* |
| Was the head posture according to SOP? |
| Were the arm(s) too close to body (touching or shadowing the torso)? |
| Were the leg(s) too close together (touching or shadowing each other)? |
| Was the hand posture according to SOP (thumbs foreward vs. towards the body) |
| Is the point cloud complete? |
| Can the waist measures be used for analysis? |
| Can the hip measures be used for analysis? |
| Any other remarks? |
|  |

SOP: standard operating procedure
